# Determination of Butyrate Synthesis Capacity in Gut Microbiota: Quantification of *but* Gene Abundance by qPCR in Fecal Samples

**DOI:** 10.3390/biom11091303

**Published:** 2021-09-02

**Authors:** Nikola Daskova, Marie Heczkova, Istvan Modos, Petra Videnska, Petra Splichalova, Helena Pelantova, Marek Kuzma, Jan Gojda, Monika Cahova

**Affiliations:** 1 Institute for Clinical and Experimental Medicine, Videnska 1958, 140 21 Prague 4, Czech Republic; mahz@ikem.cz (M.H.); modi@ikem.cz (I.M.); moca@ikem.cz (M.C.); 2 First Faculty of Medicine, Charles University, Katerinska 1660/32, 121 08 Prague, Czech Republic; 3 RECETOX, Faculty of Science Masaryk University, Kamenice 753, 625 00 Brno, Czech Republic; petra.videnska@recetox.muni.cz (P.V.); splichalova@recetox.muni.cz (P.S.); 4 Institute of Microbiology, AS CR, Videnska 1083, 142 20 Prague 4, Czech Republic; pelantova@biomed.cas.cz (H.P.); kuzma@biomed.cas.cz (M.K.); 5 Department of Analytical Chemistry, Faculty of Science, Palacky University Olomouc, 17. Listopadu 1192/12, 779 00 Olomouc, Czech Republic; 6 Department of Internal Medicine, Kralovske Vinohrady University Hospital and Third Faculty of Medicine, Charles University, Srobarova 1150, 100 34 Prague 10, Czech Republic; jan.gojda@lf3.cuni.cz

**Keywords:** gut microbiota, butyrate, functional capacity

## Abstract

Butyrate is formed in the gut during bacterial fermentation of dietary fiber and is attributed numerous beneficial effects on the host metabolism. We aimed to develop a method for the assessment of functional capacity of gut microbiota butyrate synthesis based on the qPCR quantification of bacterial gene coding butyryl-CoA:acetate CoA-transferase, the key enzyme of butyrate synthesis. In silico, we identified bacteria possessing *but* gene among human gut microbiota by searching *but* coding sequences in available databases. We designed and validated six sets of degenerate primers covering all selected bacteria, based on their phylogenetic nearness and sequence similarity, and developed a method for gene abundance normalization in human fecal DNA. We determined *but* gene abundance in fecal DNA of subjects with opposing dietary patterns and metabolic phenotypes—lean vegans (VG) and healthy obese omnivores (OB) with known fecal microbiota and metabolome composition. We found higher *but* gene copy number in VG compared with OB, in line with higher fecal butyrate content in VG group. We further found a positive correlation between the relative abundance of target bacterial genera identified by next-generation sequencing and groups of *but* gene-containing bacteria determined by specific primers. In conclusion, this approach represents a simple and feasible tool for estimation of microbial functional capacity.

## 1. Introduction

The gut microbiota is now recognized as a “new organ”, and its role in health and disease has become widely acknowledged. Emerging evidence supports the hypothesis that gut microbiota dysbiosis is closely related to the development of non-communicable diseases, including cardiovascular diseases, colorectal cancer, obesity, or type 2 diabetes (T2D) [1,2,3,4,5]. Host–microbiome interactions are heterogeneous and multifaceted, some of them being mediated by microbial fermentation products.

Short-chain fatty acids (SCFA), acetate, butyrate, and propionate, are primary products of microbial fermentation of dietary fiber [6,7,8] in the colon. Acetate is the most abundant product of fiber fermentation and serves as a suppressor of adipocyte lipolysis. It has been proposed to stimulate leptin secretion in adipocytes [9] and may also regulate appetite and satiety [10]. Propionate is metabolized in the liver, where it seems to be the inhibitor of de novo lipogenesis as well as synthesis of cholesterol [11]. Major attention is focused on butyrate. In contrast to propionate and acetate, which are transported to systemic circulation, butyrate is predominantly used by colonocytes, where it serves as a major source of energy. In addition to this trophic function, it exerts several other beneficial effects. It helps to control malignant transformation of colonocytes; in healthy cells, it promotes proliferation, while in transformed cells, it induces apoptosis [6,9,12,13,14]. Butyrate ameliorates inflammation as it binds GPR109a receptor on dendritic cells associated with intestinal mucosa and stimulates production of IL-10 and subsequent activation of anti-inflammatory Treg cells [15]. Activation of GPR109a receptor also suppresses production of pro-inflammatory cytokines TNFα and IL-6 [16]. Butyrate, as well as other SCFAs, stimulates endocrine L-cells to release GLP-2, which regulates the expression of tight junction proteins essential for maintaining intestinal barrier integrity [17]. Furthermore, butyrate stimulates MUC2 gene expression and affects mucus production [18]. Butyrate and propionate contribute to the regulation of energy homeostasis and eating behavior of the host via binding to GRP41/43 receptors, which, in turn, stimulates the production of GLP-1 and peptide YY [19].

The knowledge of functional capacity of gut microbiota is essential for personalized medicine, allowing for efficient targeted treatment tailored to a particular patient’s needs. For example, dietary fiber is often recommended due to its potential to stimulate production of SCFAs. Nevertheless, very high variability in individual response to fiber supplementation exists [20]. This variability could be explained, at least partly, by the different representation of key species of microorganisms and/or absence of the whole functional microbial communities responsible for fiber fermentation [21]. Therefore, the quantitative assessment of gut microbiota functional capacity is of great interest not only in a research context but also in terms of personalized medicine and nutrition.

The current microbiome research was enabled by the development of next generation sequencing (NGS) methods that allow sequencing of a wide variety of samples in a short time and for a reasonable price. At present, there are two main approaches to the microbiome composition determination—16S rRNA gene sequencing and shotgun sequencing [22,23]. Both methods face some limitations. Shotgun sequencing provides full information about the DNA sequence of the tested sample, i.e., DNA isolated from feces, and it is possible to derive full information about the bacteria functional capacity from the data. On the other hand, the routine implementation of this method is prevented by still relatively high costs and by high demands on bioinformatics capacity.

The analysis of variable regions of 16S ribosomal RNA gene (16S rRNA gene sequencing) is the most popular method for determination of microbial communities originating from various niches. At the end of July 2021, there were 63,875 results in PubMed search engine [24] for key word “16S rRNA sequencing”. This method is widely available and able to provide results quickly and for a reasonable price, but its main disadvantage is insufficient taxonomic resolution, which can be problematic if a deep evaluation of species or strains is needed. It is inevitable that some species are overlooked or wrongly identified, which may significantly compromise the prediction of microbiota functional capacity and result in the disagreement with conclusions derived from metatranscriptomic or metabolomic analyses.

In this study, we focused on the development of a method for the assessment of functional capacity of a selected process, in our case, butyrate synthesis in gut microbiota. Gut microbes produce butyrate through two main pathways, the butyryl-CoA:acetate CoA-transferase pathway (*but*) and the butyrate kinase (*buk*) [25,26]. Using the collection of colonic isolates obtained from healthy individuals, Louis et al. (2004) [27] demonstrated that conversion of butyryl-CoA to butyrate catalyzed by butyryl-CoA:acetate CoA-transferase (*but*) is a dominant route for butyrate formation in the human colonic ecosystem while butyrate kinase/phosphotransbutyrylase pathway was present only in a minor portion of isolates. Therefore, we developed a method based on the real-time qPCR quantification of the bacterial gene coding butyryl-CoA:acetate CoA-transferase in DNA isolated from human feces. We further show the application of this method on populations with different metabolic phenotypes, i.e., vegans and obese omnivores. Each participant signed an informed consent to the study.

## 2. Materials and Methods

### 2.1. Description of the Study Population

In this study, we used fecal samples obtained from self-reported vegans (VG, *n* = 63) who avoided all animal products for at least three years and healthy obese omnivores (OB, *n* = 62) without any dietary restrictions. In VG group, median BMI was 21.6 (min 17.6; max 32.5), in OB group, median BMI was 30.9 (min 23.3; max 55.1). VG group was comprised of younger subjects (median 31; min 18; max 58 years) compared to OB group (median 51; min 21; max 66 years). In both groups, the exclusion criteria were: age under 18 years, chronic diseases related to glucose metabolism, diseases of the digestive tract, antibiotic therapy in the past three months, pregnancy, any chronic medication (excluding hormonal contraception), and regular alcohol consumption. All data were obtained within an observational study TRIEMA supported by grant no. NV18-01-00040 MH CR. The research protocol was approved by the Ethics Committee of the Third Faculty of Medicine of the Charles University and the Ethics Committee of University Hospital Kralovske Vinohrady (EK-VP/26/0/2017) in accordance with the Declaration of Helsinki.

### 2.2. Fecal Samples Handling and Storage

Fecal samples collected at home were immediately stored at −20 °C until transported in the frozen state (<0 °C) to the laboratory within 7 days from collection. The samples were stored at −50 °C until processed. Once thawed on ice, 5–10 g of the samples were diluted in sterile water (1:4) and then homogenized using stomacher (BioPro, Prague, Czech Republic). One aliquot was used for DNA extraction, one aliquot was used for dry mass estimation, and the rest was aliquoted and stored at −50 °C.

### 2.3. DNA Isolation from Fecal Samples

DNA isolation was performed immediately after thawing and homogenization of the sample using QIAmp PowerFecal DNA Kit (Qiagen, Hilden, Germany). For each sample, two DNA isolations (each using 600 µL of the homogenate) were performed and DNA yields were combined. Then, DNA from every fecal sample was diluted to 10 ng/µL and used further on.

### 2.4. Preparation of Spike DNA

**Figure 1:**
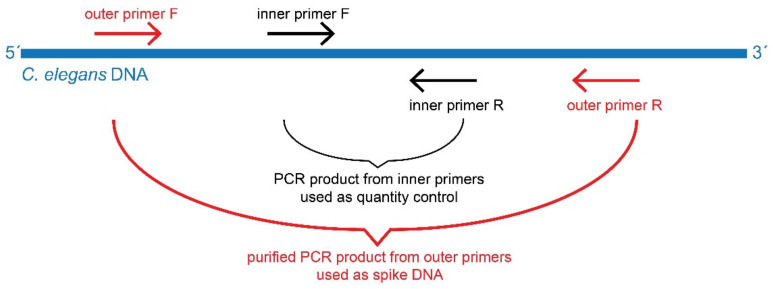
Graphical representation of spike DNA preparation.

*Caenorhabditis elegans* worms were grown on agar plates that were covered with *Escherichia coli* and harvested as described in [28]. DNA was extracted as described above. Primers for UNC-6 gene (“inner primer”) and a wider region of *C. elegans* DNA containing fragment transcribed from UNC-6 (referred to as “outer primer”) were designed in Primer 3 software [29]. The preparation of spike DNA fragment is shown on Figure 1. Sequences for both primer pairs are shown in Table 1. Using Nucleotide BLAST tool [30], we confirmed that UNC-6 gene target sequence is unique and was not found in any organism other than *C. elegans*. Using the outer primer pair, we amplified the DNA fragment containing UNC-6 sequence and separated it by gel electrophoresis. The resulting fragment was eluted using Gel Extraction Kit (Qiagen, Hilden, Germany). We obtained a fragment of *C. elegans* DNA allowing for the “inner” UNC-6 primers annealing. *C. elegans* DNA was diluted to 2 ng/µL, and after optimization, the fragment was used further on as a spike to our samples (sample DNA was mixed with *C. elegans* DNA).

### 2.5. Design of the Degenerate Primers for but Gene and Analysis of PCR Products

Degenerate primers targeting *but* gene were designed as described in Section 3. The resulting products were analyzed on Fragment Analyzer 5200 (Agilent, Santa Clara, CA, USA) using Agilent dsDNA 910 reagent Kit (35–1500 bp). The results were processed and checked in PROSize software (version 4.0.1.4).

### 2.6. qPCR

The copy number of *but* gene in DNA isolated from stool samples was determined by quantitative PCR (qPCR). qPCR reaction was performed with Syber Green master mix solution (Quanti-Tect, Qiagen, Hilden, Germany) in a volume of 20 µL with primer concentration varying from 200 nM to 1 µM (according to degeneracy number of each primer pair). Final concentration of stool DNA was 0.5 ng/µL and *C. elegans* DNA 0.1 ng/µL in the reaction mixture. Reaction was run on ViiA7 Real-Time PCR System with 96-well plates (Applied Biosystems, Waltham, MA, USA) according to the following protocol: (i) initial denaturation: 95 °C, 12 min; (ii) propagation (40 cycles): denaturation 95 °C, 15 s; annealing 60 °C, 30 s; elongation 72 °C, 20 s. The results were analyzed by SDS software version 2.3 (Applied Biosystems, Waltham, MA, USA). The copy number of genes of interest was normalized to spike DNA (*C. elegans* UNC-6 gene) or to the 16S rRNA gene (forward primer ACACTGACGACATGGTTCTACAGAGTTGATCNTGGCTCAG, reverse primer TACGGTAGCAGAGACTTGGTCTGTNTTANGCGGCKGCTG) and calculated using ΔCt method.

### 2.7. Gut Microbiome Taxonomic Analysis

DNA from fecal samples was isolated as mentioned before and V4 region of the bacterial 16S rRNA gene was amplified by PCR [31]. Sequencing analysis was performed on MiSeq (Illumina, Hayward, CA, USA) as described previously [32]. Raw sequences were processed using an in-house pipeline based on DADA2 amplicon denoiser [33]. Raw sequences were processed using standard bioinformatic procedures within the QIIME 1.9.1 package [34].

### 2.8. Quantification of Butyrate in Fecal Samples by NMR Spectroscopy

Fecal extracts for NMR analysis were prepared from homogenized stool aliquots corresponding to 1.5% of dry mass. NMR experiments (1D-NOESY and *J*-resolved with presaturation) were performed on a Bruker AVANCE III 600 MHz spectrometer (Bruker, Billerica, MA, USA) at 25 °C according to the standard protocols [35]. Butyrate signals were identified by the comparison of proton chemical shifts with HMDB database. Butyrate was quantified from 1D projections of *J*-resolved spectra to overcome the problem of signal overlap. The concentration was expressed as PQN normalized intensity of butyrate signal at 0.90 ppm.

### 2.9. Statistical Evaluation

All statistical analyses were performed using R software version 4.1.0 with in-house scripts [36]. The normality of distribution of the *but* gene abundance was tested by the Shapiro–Wilk test for normality using the shapiro.test function from the stats package (on each phylogenetically related bacteria and subject group), where the null hypothesis corresponds to data normality. Because the normal data distribution was not confirmed (*p* < 0.05), univariate statistical analyses were performed by Mann–Whitney–Wilcoxon test using the wilcox.test function from the stats package with significance of 0.05. The microbiome data were treated as compositional (proportions of total read count in each sample, nonrarefied) and, prior to all statistical analyses, were transformed using centered log-ratio transformation [37]; zero values were handled using count zero multiplicative replacement (using the cmultRepl function from the zCompositions package). The correlation between variables was assessed by using Spearman’s rank correlation coefficient utilizing cor and corrplot functions. Bland–Altman plots were used to analyze the difference between the two qPCR normalization methods (using the blandr.draw function from the blandr package).

## 3. Results

### 3.1. Identification of the Target Bacteria

As the first step, we identified bacteria containing the *but* gene (coding the enzyme butyryl-CoA:acetate CoA-transferase) in their genome using FunGene Database [38]. Every bacterium possessing this gene according to the FunGene database was searched for individually through available literature. If the bacterium was found in human gut microbiota and if it was previously confirmed as a butyrate producer [7,26,39,40], we used the bacterium further on. Every other bacterium was disregarded. In total, we identified thirty-six bacterial genomes possessing the *but* gene and meeting criteria mentioned above. Next, the specific gene sequence, as well as the amino acid sequence of the enzyme in a particular genome, was cross checked in National Centre for Biotechnology Information (NCBI) database and used further on (Table 2).

### 3.2. Design of the Degenerate Primers for but Gene

**Figure 2:**
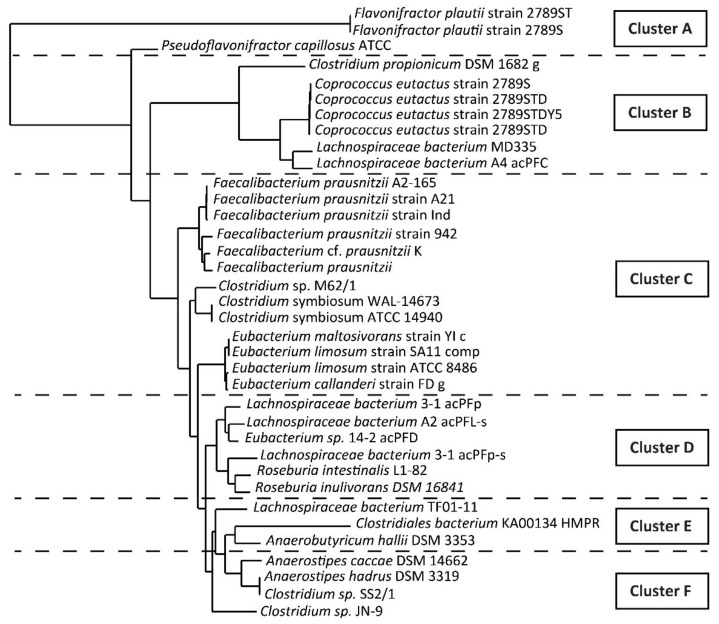
Results obtained from phylogenetic analysis. Six clusters were constructed (cluster A–F) based on phylogenetic distance. For each cluster, one pair of degenerate primers was designed.

The sequence of *but* gene coding for butyryl-CoA:acetate CoA-transferase is highly variable among gut butyrate producers, and therefore, it is not possible to design one primer fitting all target species [39,41]. Before designing any primer, the CLUSTALW tool [42] was used for a multiple sequence alignment. The diversity of the *but* coding sequence did not allow us to design one, even degenerate, primer. Therefore, we grouped the bacteria according to their phylogenetic distance, aiming to obtain groups of more similar target sequences. We used the bioinformatic web service Phylogeny.fr [43] and obtained a phylogenetic tree of closely related bacteria (Figure 2). We used the following tools: (i) MUSCLE 3.8.31 for the alignment, (ii) Gblocks 0.19b for the alignment refinement, (iii) PhyML 3.1/3.0 aLRTFor for utilizing the phylogeny analysis itself, and (iv) TreeDyn 198.3 to display the tree.

Based on the phylogenetic distance, we constructed bacterial clusters and tried to design one degenerate primer for each of the suggested clusters. If we did not succeed, i.e., the variability within the group was too high, we stepped down in the phylogenetic tree to the nearest lower cluster and designed a new set of primers. Finally, we obtained six clusters (A–F) (Figure 2). For each cluster, one pair of degenerate primers was designed by using CEMASuite software (version 2.0.9) [44]. Primers were checked with Primer BLAST tool [45] to avoid any undesired cross reactivity with other gut bacteria or human DNA. The sequences and expected lengths of the degenerate primers are shown in Table 3.

### 3.3. Validation and Optimization of Designed Primers

**Figure 3:**
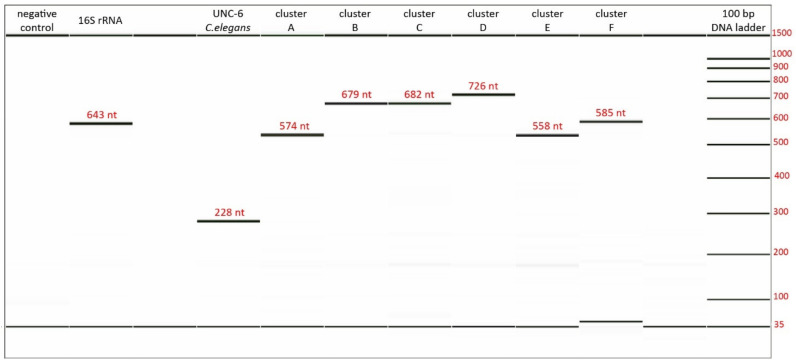
Results obtained from fragment analysis. Above every band, expected product length (number of nucleotides) is shown in red. The actual length can be estimated by the DNA ladder on the right.

Primers designed in silico were validated and optimized in DNA isolated from human stool samples. Compared to the conventional primers that perfectly match with the target sequence, the use of degenerate primers brings specific issues that must be addressed. First, a degenerate primer represents a mix of possible nucleotide combinations that do not bind to the target sequence with the same efficiency. The more degenerate the primer is, the more specific combinations may exist, and the binding efficiency to the particular target sequence is lower. Second, individual batches of the same primers are not identical, i.e., the exact combination of possible variants is unique for every single batch. Therefore, we had to experimentally define the optimal concentration of each primer for every new batch. In contrast to the usual concentration of a conventional primer (200 nM) the required concentration of degenerate primers is higher and varied in the range 200 nM–1 µM in our experiments based on the degeneracy number of each primer pair. The specificity of primer–target interaction was checked by PCR product analysis. The length of PCR products was checked with separation resolution as good as 3 bp. For all products, the predicted and determined length agreed (Figure 3).

### 3.4. Normalization of qPCR Results

**Figure 4:**
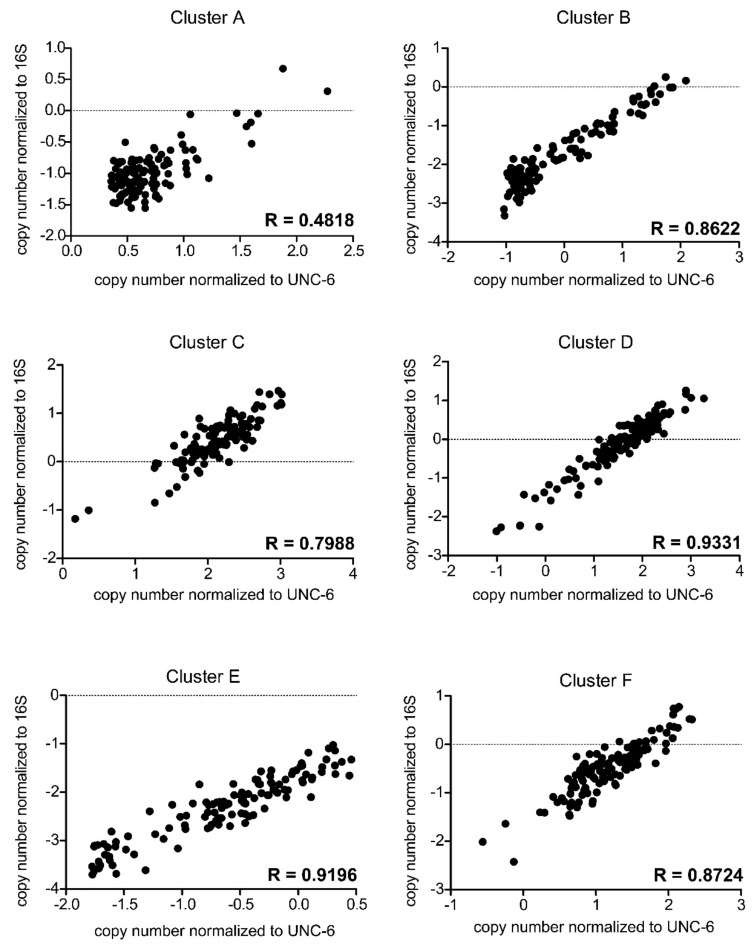
Correlation plots (each for cluster **A**–**F**) showing two different normalization methods for each cluster. *x*-axis: log transformed *but* copy number normalized to UNC-6 gene from *C. elegans*. *y*-axis: log transformed *but* copy number normalized to 16S rRNA gene. Spearman’s R correlation values are shown.

Quantification of any target sequence by qPCR depends on the stable and robust reference (housekeeper) gene. The normalization of qPCR results in stool samples is challenging as, in this material, no housekeeper gene exists. To solve this problem, we employed two different strategies. The first is based on the spike DNA, added in a standard amount to the sample prior the isolation of DNA. To this end, we chose gene coding of the protein netrin UNC-6 from *C. elegans*, as there is minimal chance of *C. elegans* natural occurrence in the human gut. The second strategy quantifies the target gene in relation to the number of 16S rRNA gene copies, particularly the conserved sequence in the V1–V3 region. Despite the differences between the normalization methods, their outcomes correlate, ranging from 0.4818 to 0.9331 of Spearman’s R values (Figure 4), *p* < 0.001.

**Figure 5:**
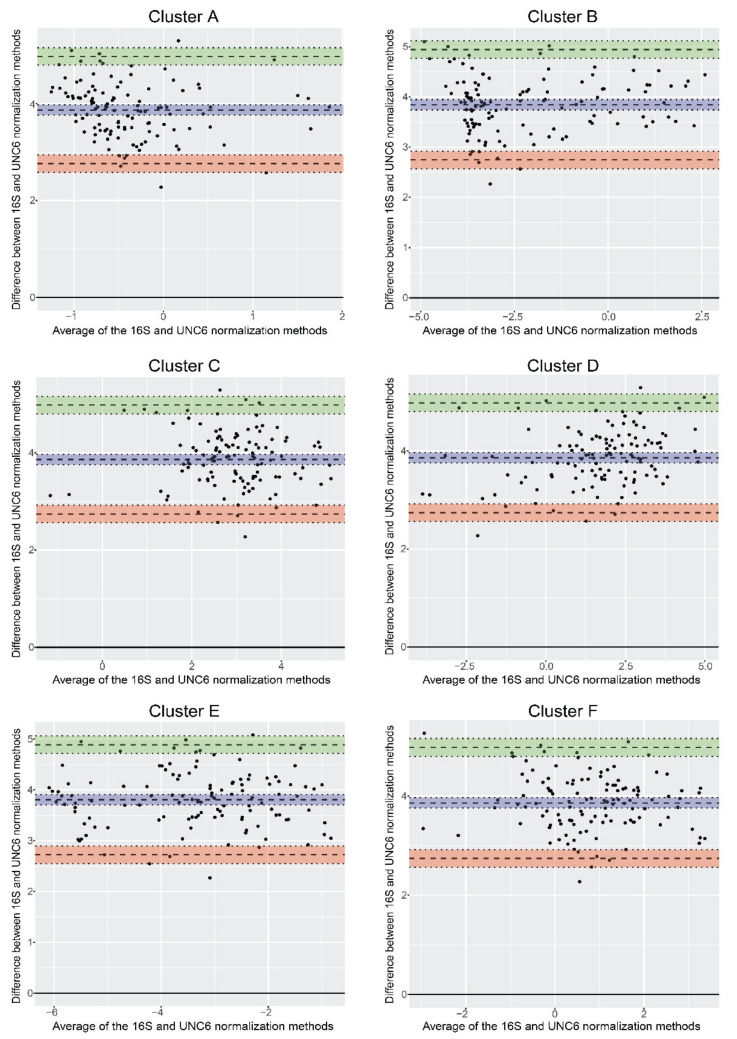
The Bland–Altman plots for each cluster that compare normalization methods based on the 16S rRNA or UNC-6 genes (after log-transform). The *x*-axis is the average copy number obtained by both normalization methods and the *y*-axis represents the difference between the outcomes of normalization according to 16S rRNA and UNC-6 genes. The blue region is the bias with its 95% confidence interval, green region is the upper limit of agreement with its 95% confidence interval, and the red region is the lower limit of agreement with its 95% confidence interval.

Nevertheless, significant correlation between the outcomes of two ways of *but* gene abundance normalization does not prove that these two methods are identical. Therefore, we employed the Bland–Altman method (Figure 5). The analysis revealed that the ratio between the normalization methods does not change with the magnitude of the measured values. The results of correlation and Bland–Altman analyses revealed comparable outcomes from both normalization methods.

### 3.5. Quantification of but Gene in Populations with Contrast Phenotypes

We determined the abundance of the *but* gene in two experimental groups with different dietary habits and metabolic phenotypes, i.e., lean vegans and obese omnivores. In both vegans and omnivores, we found the highest abundance of the *but* gene was determined by primers specific for cluster C and lowest using those specific for cluster B (B < E < A < F < D < C). As shown in Table 4, vegans and obese omnivores significantly differed in the *but* gene abundance in cluster A (higher in OB) and cluster C (higher in VG). *But* gene copy number in cluster D tended to be higher in VG as well, but it did not reach statistical significance. Cluster A comprises *Flavonifractor plautii* and *Pseudoflavonifractor capillosus*. Cluster C encompasses *Faecalibacterium prausnitzii*, *Clostridium symbiosum*, *Clostridium* sp. M62/1, and three species belonging to genus *Eubacterium*. Cluster D is comprised of three species belonging to genus *Lachnospiraceae*, *Roseburia intestinalis* and *Roseburia inulivorans,* and one *Eubacterium* species. For quantification, we used both normalization methods. The results were comparable in terms of abundance of individual clusters and VG to OB ratio. In absolute values, the obtained numbers were lower using 16S rRNA gene for normalization due to the high abundance of this gene compared with the gene of interest (*but*). The difference between both normalization methods lies in the unstable 16S rRNA gene copy number per bacterial genome at various bacterial taxa. However, as shown by the downstream-statistical analysis (Table 4), both normalization methods were able to detect the same significant or insignificant differences between the subjects.

**Figure 6:**
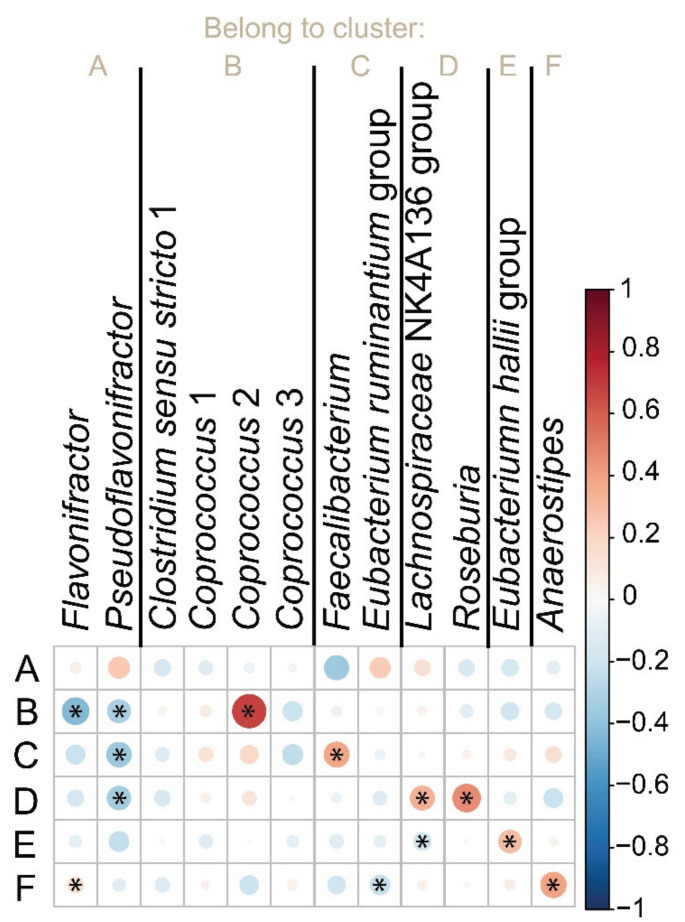
Graphical representation of Spearman’s correlation matrix between *but* copy number identified by primers A–F and relative abundance of representative bacteria for every cluster determined by NGS. The *but* copy number was normalized to UNC-6 gene. The circle size and color intensity are proportional to Spearman’s coefficient value. The red color of a circle indicates positive correlation, the blue color negative correlation. Areas with an asterisk sign inside the circle indicate that the specific correlation was significant on the significance level of 0.05.

**Figure 7:**
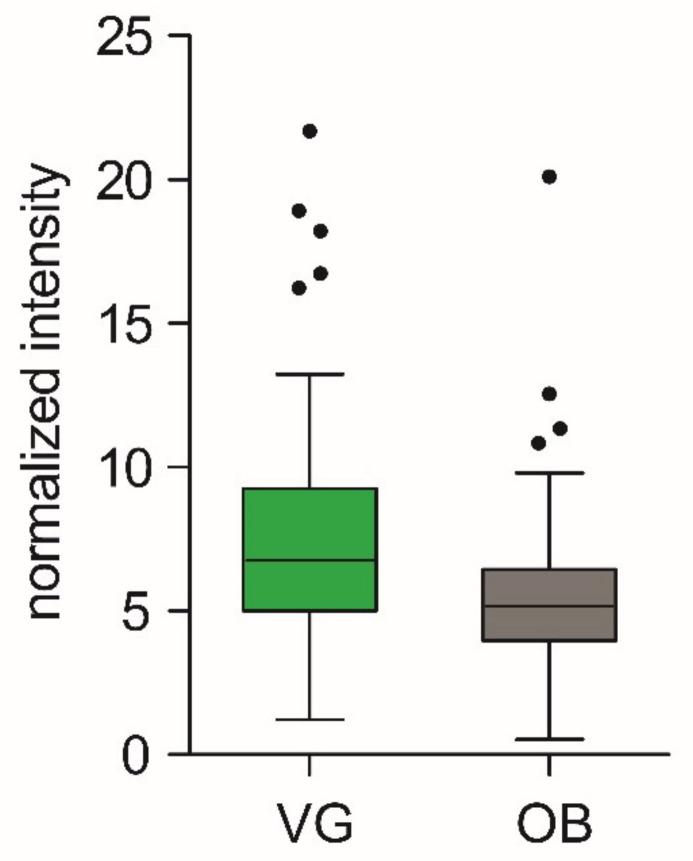
Butyrate content in feces of VG and OB subjects. Values represent PQN normalized intensities of butyrate-specific signal in NMR spectra. Data are presented as Tukey box plots with median and whiskers (1st, 3rd quartile).

Aiming to validate these results, we correlated the abundance of *but* gene in different clusters with the microbiome composition of the same samples (Supplementary Table S1) determined by 16S rRNA gene sequencing (Figure 6). In the cases of clusters B, C, D, E, and F, the abundance of *but* gene most significantly correlated with the expected bacterial taxa (significance level was set to 0.05). Regarding cluster A, we found a trend to the correlation between the abundance of gene *but* and *Pseudoflavonifractor* and, to a lesser extent, *Flavonifractor*, but these correlations were not significant on the chosen significance level. Importantly, our findings, i.e., higher copy number of *but* gene in vegan group, were in line with the significantly higher amount of butyrate in vegan fecal samples, *p* = 0.002 (Figure 7).

## 4. Discussion

In this study, we describe the development of a widely accessible method for the assessment of functional capacity of gut microbiota for butyrate synthesis based on the qPCR quantification of bacterial butyryl-CoA:acetate CoA-transferase. The workflow includes: (i) isolation of DNA from the fecal sample; (ii) performing multiple qPCRs using degenerate primers specific for *but* gene variants; and (iii) quantification of *but* gene abundance using the selected reference gene. We further demonstrate the application of this method in the assessment of butyrate synthesis capacity in the fecal microbiome of lean vegans and healthy obese omnivores. Our results show that some *but* gene variants are far more abundant than the others; therefore, when the aim is only the approximate estimation of butyrate synthesis capacity, the method could be further simplified by using only the primers specific for the most abundant clusters of bacteria.

Quantification of a particular gene of interest (GOI) in the studied microbial population allows for deeper insight into the real functional capacity of the particular sample. On the other hand, this approach must cope with another type of limitations. Variability of GOI sequences among bacteria does not allow for designing one universal primer. This obstacle could be overcome by employing degenerate primers, but even adopting this approach, it is usually impossible to design one primer for all target sequences. We propose that the identification of phylogenetically related groups of bacteria sharing some similarity is the solution to this problem. Because of the random selection of nucleotides in the degenerate positions, individual batches of degenerate primers may significantly vary in composition. This inconvenience could be overcome by preparation of well-defined primer mixtures where each primer pair is present in the exact concentration. In practice, each primer pair may be synthesized separately and then all primers mixed in equimolar ratio. This approach may be more expensive and laborious at the beginning, but it allows for avoiding the necessity to determine the efficient primer concentration for every new batch.

The qPCR method is comparative in principle. Therefore, the selection of a stable reference gene is of utmost importance. Unfortunately, feces is quite challenging material from this point of view. It is extremely heterogeneous and varies greatly in the content of dry mass, total protein or total DNA. A standardized fecal sample processing and DNA isolation pipeline during the study is essential, as it turns out in recent years [46,47]. Furthermore, DNA isolated from feces is a mix of host, bacteria, fungi, and virus DNA at variable ratios. The final readout, i.e., the composition of the fecal microbial community, is affected by the stabilization and storage strategies used in the process of sampling. The most important factors are: (i) usage of preservation buffers; (ii) time from sample production and freezing; (iii) storage temperature, and (iv) aerobic vs. anaerobic conditions during storage [48]. A standardized fecal sample processing and DNA isolation pipeline during the study is essential, as it turns out in recent years [46,47]. In our study, we decided for sample storage without preservation buffers, as their presence makes subsequent metabolomic analysis impossible. The native samples were immediately frozen in −20 °C for maximum 7 days; long-term storage occurred at −50 °C. Because the quick-freezing stops or maximally slows down biological processes, we processed the samples under aerobic conditions, as it simplifies the sample handling for the study participants.

The most commonly known gene shared by all bacteria and, thus, the potential housekeeper gene is the gene coding for 16S rRNA. Its advantage as a reference gene is that it contains conserved sequences common to all bacteria that could serve as target sequences of primers. The quantification of the 16S rRNA gene allows for the assessment of the total bacterial “load” in the sample. The limitation of 16S rRNA gene as a reference is the unequal number of 16S rRNA copies per cell in different bacterial species.

According to Větrovský and Baldrian’s in silico study [49], with a total number of 1690 bacterial genomes with 909 species identified, there was an average of 4.2 16S rRNA gene copies per genome. The copy number of 16S rRNA gene was highly taxonomically specific (for example, in the Gammaproteobacteria and Fusobacteria, the copy number varied widely between 1 and 15). In some studies [25], the 16S rRNA gene copy number was used for normalization of the *but* gene but had to be adjusted as an average number of copies in particular bacteria; in this case, five gene copy numbers were used representing Firmicutes and Bacteriodetes. Second, the abundance of 16S rRNA gene is an order of magnitude higher than the abundance of GOI, which results in low absolute values of the GOI copy number normalized to this reference gene.

An alternative possibility of normalization in this type of material is quantification to external DNA added to the sample. This approach is independent of microbiome composition of the sample, and it may serve as a quality control of the whole process as well. The prerequisite is the choice of sequence that is not present in any organism, which could be found in the target material, in our case, in human feces. The UNC-6 gene of *C. elegans* was a good candidate as we did not find any corresponding sequence when using Nucleotide BLAST tool [30]. Despite the limitations of both approaches, we observed high correlation between the *but* gene copy number normalized to both 16S rRNA and UNC-6 sequences, and Bland–Altman analysis revealed the similarity of the outcomes of both normalization methods. Nevertheless, we should be always aware of different numbers of 16S rRNA gene copies within the bacterial population. Therefore, we prefer the normalization to external DNA added to the sample; 16S rRNA gene quantification can be used for the control of equal loading of bacterial DNA to the PCR reaction.

The rationale behind this study was to develop a widely accessible and easily implementable method that would allow for the assessment of the functional capacity of the gut microbiota. The utmost readout of metabolic performance of bacteria is the presence and quantity of the particular metabolite(s). In the case of the *but* gene, such a readout is the content of butyrate in feces. Vegans and omnivore subjects represent different phenotypes in terms of gut microbiota and metabolome composition [23,50,51,52]. Furthermore, a vegan diet (as well as other plant-based diets) is associated with a high production of butyrate by bacteria in the colon [50]. Based on these presumptions, we hypothesized that the *but* gene will be more expressed in vegan compared to omnivore microbiota.

Based on the phylogenetic distance, we designed six sets of degenerate primers (marked as cluster A–cluster F), every primer set targeting a different cluster of bacteria possessing the *but* gene. It was confirmed that the *but* gene was more expressed in vegans for cluster C, which encompasses abundant genera *Faecalibacterium*, *Clostridium,* and *Eubacterium*, which belong to the known butyrate producers, and *but* gene copy number detected by primers specific for this cluster was highest among all clusters tested. We also found a trend of higher *but* gene copy numbers in vegans detected for cluster D. Cluster D includes rather abundant butyrate producers *Lachnospiraceae bacterium*, *Eubacterium*, *Roseburia intestinalis,* and *Roseburia inulivorans*. Based on these results, we predicted higher butyrate production in vegans, which was confirmed by NMR analysis of butyrate content in fecal samples. Using two different approaches, the determination of *but* gene abundance in fecal DNA and direct assessment of butyrate content in feces, we confirmed the higher butyrate production capacity in vegans. This finding supports the feasibility of our method in predicting the microbial functional capacity.

As we had to employ primers specific to defined groups of bacteria, our method may provide additional information about microbiota composition. Aiming to verify this assumption, we calculated the correlation of *but* gene copy number and microbiota composition in the same sample determined by 16S rRNA sequencing. For clusters B, C, D, E and F we found strong positive correlation between the *but* gene copy number and the abundance of the target bacteria. In these clusters, the primer sequences are derived from abundant and/or highly prevalent bacteria, such as *Coprococcus* (cluster B), *Faecalibacterium* (cluster C), *Roseburia* (cluster D), *Eubacterium/Anaerobutyricum hallii* (cluster E), or *Anaerostipes* (cluster F). In cluster A, we observed a weak positive correlation (not a significant one) between *but* gene copy number and the abundance of target bacteria *Pseudoflavonifractor* and, to a lesser extent, *Flavonifractor*. Both these bacteria were identified in our sample set but with low abundance and prevalence, which might compromise the outcome of our method.

## 5. Conclusions

Taken together, we described a method allowing for the detection of specific bacterial genes in the gut microbiome. Our data support the presumption that the determination of *but* gene copy number on bacterial DNA reflects its taxonomic composition, particularly in the case of more abundant bacteria, as well as functional readout, in this case fecal butyrate content. In conclusion, this approach may represent an efficient tool for the estimation of microbial functional capacity. This method requires only equipment and skills routinely available in diagnostic laboratories and does not put any demands on advanced bioinformatics data analysis. Therefore, it may become a feasible tool for rapid screening of specific functional capacity of gut microbiota, i.e., allowing for personalized estimation of the usefulness of prebiotic treatment.

## Figures and Tables

**Table 1:** Sequences of inner and outer primer pairs used in spike preparation.

Primer Name	Primer Sequence (5′ to 3′)
inner primer forward	GAAGAGCAAGATCAGTGTTC
inner primer reverse	CTTGCAAATGACACCTTG
outer primer forward	GTAATCGTTGTGCCAAAGG
outer primer reverse	TCCTCCCATTCCACCAATAC

**Table 2:** Butyrate producers in human gut microbiota possessing the *but* gene. A total of thirty-six *but* gene nucleotide and protein sequences were identified (stated as an NCBI accession numbers).

Taxonomy (including the Strain)	Nucleotide Sequence Accession Number	Protein Sequence Accession Number
*Anaerostipes caccae* DSM 14662	ABAX03000012	WP_006566634
*Anaerobutyricum hallii* DSM 3353	ACEP01000025	EEG37758
*Anaerostipes hadrus* DSM 3319	AMEY01000089	EKY19441
*Clostridiales bacterium* KA00134	LTAF01000006	KXO16903
*Clostridium* sp. JN-9	CP035280	QAT39812
*Clostridium* sp. M62/1	ACFX02000051	EFE10856
*Clostridium* sp. SS2/1	ABGC03000034	EDS21983
[*Clostridium*] *propionicum* DSM 1682	FQUA01000004	SHE65336
[*Clostridium*] *symbiosum* ATCC 14940	AWSU01000039	ERI80067
[*Clostridium*] *symbiosum* WAL-14673	ADLR01000107	EGB17928
*Coprococcus eutactus* 2789STDY5608829	CYYZ01000002	CUN77211
*Coprococcus eutactus* 2789STDY5608843	CYYJ01000005	CUO17024
*Coprococcus eutactus* 2789STDY5608888	CYYE01000001	CUN69525
*Coprococcus eutactus* 2789STDY5834963	CYXU01000007	CUN05838
*Eubacterium callanderi* FD	FRBP01000012	SHM18802
*Eubacterium limosum* ATCC 8486	CP019962	ARD67787
*Eubacterium limosum* SA11	CP011914	ALU15403
*Eubacterium maltosivorans* YI	CP029487	QCT73558
*Eubacterium* sp. 14-2	ASSS01000012	EOT23498
*Faecalibacterium prausnitzii* 942/30-2	CP026548	AXA81262
*Faecalibacterium prausnitzii* A2165 CG447_00005	CP022479	ATO98751
*Faecalibacterium prausnitzii* A2-165 FAEPRAA2165_01562	ACOP02000044	EEU96797
*Faecalibacterium prausnitzii* APC918/95b	CP030777	AXB28579
*Faecalibacterium prausnitzii* Indica	CP023819	ATL89114
*Faecalibacterium prausnitzii* KLE1255	AECU01000083	EFQ07628
*Flavonifractor plautii* 2789STDY5834892	CZAS01000006	CUP57950
*Flavonifractor plautii* 2789STDY5834932	CZBD01000023	CUQ37563
*Lachnospiraceae bacterium* 3-1	ASST01000018	EOS23550
*Lachnospiraceae bacterium* 3-1	ASST01000032	EOS21051
*Lachnospiraceae bacterium* A2	ASSX01000004	EOS48506
*Lachnospiraceae bacterium* A4	ASSR01000007	EOS36856
*Lachnospiraceae bacterium* MD335	ASSW01000016	EOS51721
*Lachnospiraceae bacterium* TF01-11	LLKB01000001	KQC86641
*Pseudoflavonifractor capillosus* ATCC 29799	AAXG02000004	EDN01706
*Roseburia intestinalis* L1-82	ABYJ02000099	EEV00989
*Roseburia inulinivorans* DSM 16841	ACFY01000152	EEG92587

**Table 3:** Sequences and expected product lengths of each primer set.

Primer Name	Primer Sequence (5′ to 3′)	Expected Product Length (nt)
*but* cluster A forward	MCTGGGYATYCACACCGAG	574
*but* cluster A reverse	GGTGGGCGATGGAGATAA	
*but* cluster B forward	GGKCCBATHGARRTTGCAGA	679
*but* cluster B reverse	TKTCGTCMASCCABTCATAC	
*but* cluster C forward	GBGACTGGSTRGATTAYG	682
*but* cluster C reverse	TCVACRTACATYTCSGTGTG	
*but* cluster D forward	TGGAAYTCMTGGCATATGTC	726
*but* cluster D reverse	VGMRTTGTTRATGGAMATAAA	
*but* cluster E forward	TGHAGSABHTSWTTTTACATGGA	558
*but* cluster E reverse	SSCTTTGCAATGTCAACAAA	
*but* cluster F forward	AAATATGCCTCGHTGCYTWG	585
*but* cluster F reverse	ARRTARGCACCYAWAACGAAATC	

Primers designed for *but* gene are divided based on their phylogenetic distances into clusters A–F. nt = nucleotide; K = G or T; B = C or G or T; H = A or C or T; R = A or G; M = A or C; S = G or C; Y = C or T; V = A or C or G; W = A or T (as stated by IUPAC nucleotide code).

**Table 4:** *but* gene copy number normalized to either UNC-6 gene from *C. elegans* or to 16S rRNA gene in vegan (VG) and obese (OB) subjects. Data are given as median (IQR, interquartile range). In each sample, *but* gene copy number was determined using all primer pairs in separate qPCR reactions. The similarity of the distribution in VG and OB groups was tested using Mann–Whitney U test. The results were considered statistically significantly different at *p* < 0.05 (shown in bold).

	Cluster A		Cluster B		Cluster C		Cluster D		Cluster E		Cluster F	
	UNC	16S	UNC	16S	UNC	16S	UNC	16S	UNC	16S	UNC	16S
VG	3.5 (2.0)	0.08 (0.06)	0.45 (5.09)	0.01 (0.09)	211 (243)	4.9 (5.8)	63 (118)	1.1 (2.4)	0.28 (0.48)	0.01 (0.01)	12.5 (25.1)	0.34 (0.41)
OB	4.8 (3.5)	0.10 (0.10)	0.28 (1.91)	0.01 (0.05)	86 (71)	1.8 (1.6)	34 (76)	0.9 (1.6)	0.32 (0.61)	0.01 (0.01)	17.6 (30.9)	0.38 (0.70)
*p*-value	**0.004**	**0.019**	0.942	0.438	**<0.001**	**<0.001**	0.167	0.225	0.769	0.840	0.680	0.589

## Data Availability

Sequencing data are available at European Nucleotide Archive database under the accession number PRJEB43938.

## Supplementary Materials

The following are available online at https://www.mdpi.com/article/10.3390/biom11091303/s1, Table S1: title. Microbiome composition of the fecal samples determined by 16S rRNA gene sequencing.
